# Ovariotomy—Use of the Actual Cautery for the Division of the Pedicle

**Published:** 1867-04

**Authors:** 


Ovariotomy—Use of the Actual Cautery for the Division of the Pedicle. The remarkable success which has recently crowned this operation, where the pedicle and the adhesions have been divided by the actual cautery, seems to place the operation of ovariotomy beyond all cavil. The instrument employed by Mr. Baker Brown for this purpose is, as we understand, a wedge-shaped piece of iron, with which the division is accomplished by a sawing motion.

